# Biosynthesis, Characterization, and Biological Activities of Procyanidin Capped Silver Nanoparticles

**DOI:** 10.3390/jfb11030066

**Published:** 2020-09-19

**Authors:** Umar M. Badeggi, Jelili A. Badmus, Subelia S. Botha, Enas Ismail, Jeanine L. Marnewick, Charlene W. J. Africa, Ahmed A. Hussein

**Affiliations:** 1 Department of Chemistry, Cape Peninsula University of Technology, Symphony Rd., Bellville 7535, South Africa; 217064221@mycput.ac.za (U.M.B.); enas.ismail4@yahoo.com (E.I.); 2 Applied Microbial and Health Biotechnology Institute, Cape Peninsula University of Technology, Symphony Rd., Bellville 7535, South Africa; jabadmus@lautech.edu.ng (J.A.B.); marnewickj@cput.ac.za (J.L.M.); 3 Electron Microscope Unit, University of the Western Cape, Bellville 7535, South Africa; subotha@uwc.ac.za; 4 Department of Medical Biosciences, University of the Western Cape, Bellville 7535, South Africa; cafrica@uwc.ac.za

**Keywords:** biosynthesis, procyanidins dimers, *Leucosidea sericea*, silver nanoparticles, phytoconstituents, antimicrobial, antidiabetic, antioxidant

## Abstract

In this study, procyanidin dimers and *Leucosidea sericea* total extract (LSTE) were employed in the synthesis of silver nanoparticles (AgNPs) and characterized by ultraviolet-visible (UV-Visible) spectroscopy, high-resolution transmission electron microscopy (HRTEM), selected area electron diffraction (SAED), X-ray diffraction (XRD), and dynamic light scattering (DLS) techniques. AgNPs of about 2–7 nm were obtained. DLS and stability evaluations confirmed that the AgNPs/procyanidins conjugates were stable. The formed nanoparticles exhibited good inhibitory activities against the two enzymes studied. The IC_50_ values against the amylase enzyme were 14.92 ± 1.0, 13.24 ± 0.2, and 19.13 ± 0.8 µg/mL for AgNPs coordinated with LSTE, F1, and F2, respectively. The corresponding values for the glucosidase enzyme were 21.48 ± 0.9, 18.76 ± 1.0, and 8.75 ± 0.7 µg/mL. The antioxidant activities were comparable to those of the intact fractions. The AgNPs also demonstrated bacterial inhibitory activities against six bacterial species. While the minimum inhibitory concentrations (MIC) of F1-AgNPs against *Pseudomonas aeruginosa* and *Staphylococcus aureus* were 31.25 and 15.63 µg/mL respectively, those of LSTE-AgNPs and F2-AgNPs against these organisms were both 62.50 µg/mL. The F1-AgNPs demonstrated a better bactericidal effect and may be useful in food packaging. This research also showed the involvement of the procyanidins as reducing and capping agents in the formation of stable AgNPs with potential biological applications.

## 1. Introduction

Metallic nanoparticles are of great interest owing to their unique physicochemical characteristics as well as their potential biomedical applications [1]. The characteristic properties of metallic nanoparticles (MNPs) such as silver nanoparticles (AgNPs) depend on the methods of preparation and the nature of the precursors [1]. Physical methods [2,3] have been used to prepare AgNPs; however, they are not cost-effective, consume more energy, and involve the use of sophisticated instruments [4]. Through the chemical procedures, reducing agents such as hydrazine have been employed in the formation of MNPs [1]. Although the nanoparticles possess interesting characteristic features, they have limited biological applications due to toxicity concerns [5]. Their stability is usually enhanced with the use of external stabilizers, some of which are also toxic [6]. Using biological resources to synthesize AgNPs, among which plants are more popular [7,8,9], could eliminate the toxicity problem. Plants are readily accessible, non-toxic, and can be easily handled [10]. Plants also possess phytochemicals, serving not only as reducing, but also as capping agents, making the synthesis a facile process [11].

Several AgNPs have been successfully biosynthesized using various plant extracts such as *Nigella arvensis L* [12], *Pelargonium graveolens* [13], *Theobroma cacao* and *Vitis vinifera* seed [14], *Putranjiva roxburghii* [15], *Combretum erythrophyllum* [16], and *Ducrosia anethifolia* [17]. An array of spectroscopic and microscopic techniques have been employed to characterize the AgNPs [11,13,15,16,18]. In these extracts, phytoconstituents such as polyphenols are believed to be responsible for the reduction and stabilization of the AgNPs. To identify them, Fourier-transform infrared (FTIR) spectroscopy was employed [19] in addition to nuclear magnetic resonance (NMR) spectroscopy [20] and high-performance liquid chromatography coupled with mass detector (LC-MS) [21]. Fractions or pure natural compounds have also been used in the green synthesis of nanoparticles [22] and examples include tannic acid [23], quercetin, gallic acid [24], and other compounds [25,26]. Green synthesis is the preferred procedure for the formation of biocompatible nanoparticles [27]. However, better knowledge of the chemical composition of the extracts or fractions is necessary to further understand the role of the phytochemicals either as the reducing or capping agent or both. Moreover, little is known about procyanidins in higher plants and their reducing/capping abilities in green synthesis. This was one of the aims of the present study.

Furthermore, biosynthesized AgNPs prepared from the chemical constituents of various plant extracts and compounds have shown potent antioxidant [28,29], antidiabetic [30,31,32,33] and antibacterial [34] activities. However, further studies are necessary on these greener alternatives.

*Leucosidea sericea* Eckl and Zeyh is an evergreen shrub that belongs to the family *Rosaceae*. Nine out of its 3000 species are found in South Africa and its neighboring countries. Commonly called “old wood,” the plant grows on both dry and wetlands. It has a silvery, woody bark and can be up to 7 m in height [35]. The extract from this plant has been used to cure many ailments and has proven biological properties [36,37]. A number of compounds have been isolated from the extract of *L. sericea* and showed various bio-activities [35,38,39]. The mentioned traits awaken the curiosity to further investigate this wonder plant. Recently, our group identified procyanidins in the extract for the first time [40]. Procyanidins are a higher class of polyphenols with unique chemical structure, and mostly occur in fruits and vegetables. Their presence is, therefore, a pointer to the significance of this plant. The quality of raw materials used as food depends largely on phenolic compounds such as procyanidins [41]. Because of the abundant presence in fruits, legumes, cereals, and a variety of beverages, procyanidins represent up to 50% of dietary polyphenols we consume daily [42]. These procyanidins have displayed diverse biological activities, and their polyphenolic nature may offer them enhanced biochemical activities [43,44]. Recent studies reported an increase of bioavailability for the flavonoid epigallocatechin gallate when capped with metal NPs [45], and this may be applicable to procyanidins as well. As mentioned, the unique chemical structures of procyanidins, have also rendered them excellent reducing and capping agents [40] that have the capability to synthesize stable, safe, and bio-active metal nanoparticles with possible future medical applications. In the same context and to avoid using toxic ingredients, the present study employed highly purified procyanidin dimers and fractions for the formation of stable and bioactive AgNPs. Their potential antioxidant, antidiabetic, and antimicrobial properties were evaluated and a detailed mechanism on the involvement of procyanidins in the formation of AgNPs was proposed.

## 2. Materials and Methods

### 2.1. Materials

Polystyrene 96-well microtitre plates were supplied by Greiner bio-one GmbH (Frickenhausen, Baden-Württemberg, Germany). Silver nitrate, iron (III) chloride hexahydrate, 2,4,6-tris(2-pyridyl)-s-triazine, hydrochloric acid (HCl), alpha-glucosidase (*Saccharomyces cerevisiae*), alpha-amylase (procaine pancreas), 3,5-dinitro salicylic acid (DNS), *p*-nitrophenyl-α-D-glucopyranoside (*p*-NPG), sodium carbonate (Na_2_CO_3_), sodium dihydrogen phosphate, Ampicillin, disodium hydrogen phosphate, trolox (6-hydroxyl-2, 5, 7, 8-tetramethylchroman-2-carboxylic acid), 2,2-azino-bis (3-ethylbenzothiazoline-6-sulfonic acid) (ABTS) diammonium salt, iodonitrotetrazolium chloride (INT), potassium peroxodisulphate, gallic acid, vitamin C, and sodium chloride (NaCl) were bought from Sigma-Aldrich (Cape Town, Western Cape, South Africa). N-Acetyl-L-cysteine (CYS), glycine (GLY), and Folin-Ciocalteu’s phenol reagent (FC) were procured from Boehringer Mannheim GmbH (Mannheim, Baden-Württemberg, Germany). Phosphate buffered saline (PBS) was purchased from Lonza (Cape Town, Western Cape, South Africa). Bovine serum albumin (BSA) was procured from Miles Laboratories (Pittsburgh, PA, USA). Brain-heart infusion broth (BHI) and Mueller-Hinton Agar were purchased from Biolab (Merck, Modderfontein, South Africa). A microtitre plate reader (BMG Labtech, Ortenberg, Germany) was employed in reading the absorbance of the AgNPs and in biological studies. A high-resolution transmission electron microscopy (FEI Tecnai G2 F20 S-Twin HRTEM, operated at 200 kV) was used to study the morphology of the AgNPs. X-ray diffraction (XRD; Bruker (Billerica, MA, USA) AXS D8 advance diffractometer with CuKα1 radiation (λ = 1.5406 Å)) was employed to study the crystallinity of the particles. A Malvern Zetasizer Instrument (Malvern Ltd., Worcestershire, UK) was used for DLS examinations.

### 2.2. Extraction of Phytochemicals and Formation of SILVER Nanoparticles

#### 2.2.1. Extraction and Purification of Chemical Constituents

The aerial parts of *Leucosidea sericea* were extracted with 50% aqueous-ethanol to render the total extract (referred to as LSTE). A portion of the total extract was partitioned in ethyl acetate and subsequently purified using different chromatographic techniques [40]. Briefly, silica gel column chromatography was employed using a gradient of hexane and ethyl acetate of increasing polarity. The fractions containing procyanidins were further chromatographed on silica gel using isocratic ethyl acetate. The combined fractions from the above were then subjected to smaller column (3 × 30 cm) chromatography using sephadex using methanol/water (90:10 and/or 80–20). The fraction(s) which demonstrated a single spot on the TLC were submitted for NMR analysis to further confirm the purity. These fractions were labelled F1 and F2, and submitted for both NMR and LC-MS analyses. The spectra revealed the presence of procyanidins in F1 and F2.

#### 2.2.2. Biosynthesis of Silver Nanoparticles

The synthesis of AgNPs was done by dissolving 20 mg of the aerial part extract (LSTE), F1, or F2 in 2 mL of deionized Milli-Q water and vortexing for 5 min. The resulting yellowish solution was then added to a 70 mL of 1.0 mM silver nitrate solution at 70 °C. After 10 min, a colour change was observed from yellowish to brown, which confirmed the successful synthesis of AgNPs. The reaction was monitored with absorbance reading (300–650 nm) until no further changes were noticed. The heat was removed, and the reaction mixture stirred for another 1 h in the dark. The colloidal solutions were allowed to cool to room temperature before several washing centrifuge steps. This was done to remove any unreacted substances that may still be in the solution.

### 2.3. Stability Evaluation of AgNPs

The procedure of Elbagory et al. [46] was adopted with minor adjustments. Briefly, 0.5% (NaCl, CYS, GLY, and BSA), deionized water, and PBS (pH 7 and 9) were used. The evaluation was carried out in a 96-well plate where the colloidal solution and the freshly prepared media and buffers were added. For every 160 µL of the colloidal solution, a volume of 80 µL of the above solutions was added in a different well. This was manually agitated for proper mixing and the absorbance immediately measured. This was termed as the zero-hour reading. The plate was then covered, wrapped in aluminum foil, and incubated at 37 °C for 24 h. After 24 h, another measurement was then taken and the plate was returned to the oven for an additional 24 h. At the end of this time, the 48 h reading was taken. This process was followed for each of the three (LSTE, F1, and F2) AgNPs.

### 2.4. Dilution Study

The sample of AgNPs in the powdered form was obtained as reported by Badeggi and colleagues [40]. Briefly, 15 mL of the sample was freeze-dried in a falcon tube after several washing and centrifugation steps. 300 µL of the synthesized AgNPs were measured at different concentrations. The concentrations were also plotted alongside the intensity to understand the linearity of the readings.

### 2.5. In-Vitro Enzymatic Assay

#### 2.5.1. Alpha-Amylase Inhibitory Activity

A standard protocol was employed where 50 µL of phosphate buffer (0.01 M, pH 6.9), 20 µL of procaine pancreatic alpha-amylase (2U/mL) solution, and 20 µL of the samples were all added in a 96-well plate and incubated for 20 min at room temperature [47]. After this pre-incubation, 20 µL of 1% soluble starch was added to the mixture and incubated for an additional 30 min. This was immediately followed by the addition of 100 µL of 3,5-dinitro salicylic acid (DNS) which stopped the reaction. The reaction mixture was then incubated in a boiling water bath for 10 min before the absorbance was read at 540 nm. Acarbose was used as the standard and each experiment was done in triplicate. Equation (1) was used to calculate the percentage inhibition of the alpha-amylase activity.

(1) $$\mathrm{Percentage}\;\mathrm{inhibition}\;\left(\%\right)=\left(C-\frac{T}{C}\right)\times 100\;$$

where C and T are absorbance readings of the control and the treated sample, respectively.

#### 2.5.2. Alpha-Glucosidase Inhibitory Activity

A standard procedure of the alpha-glucosidase assay was followed with slight modification [30]. Briefly, 20 µg/mL of the three AgNP samples was serially diluted in a 96-well plate using a 100 mM phosphate buffer (PBS) at pH 6.8. The mixture was gently agitated and allowed to stand for 15 min at 25 °C. Thereafter, 20 µL of a 5 mM *p*-nitrophenyl-α-D-glucopyranoside (*p*-NPG) was added as the substrate and the plate was incubated at 37 °C for 20 min. Hereafter, 50 µL of a 0.1 M Sodium carbonate (Na_2_CO_3_) solution was added to each well to stop the reaction. With the aid of a plate reader, the absorbance measurement was then taken at 540 nm. The wells with enzyme, buffer, and substrate but without samples served as positive controls and each experiment was conducted in triplicate. The percentage inhibition of the enzymatic property of alpha-glucosidase was determined using Equation (1) in Section 2.5.1.

### 2.6. Antibacterial Activity

The protocol description of Elbagory et al. [46] was adopted with slight changes. Briefly, BHI was used for the serial dilution of the three AgNPs to include six concentrations, i.e., 125.00, 62.50, 31.25, 15.63, 7.81, and 3.90 µg/mL. The six bacterial test species *Pseudomonas aeruginosa*, *Staphylococcus aureus*, *Bacillus cereus, Salmonella enterica*, *Escherichia coli* and *Serratia marcescens* (in PBS) were standardized to 0.5 McFarland equivalents and further diluted in BHI. Three negative controls were included and consisted of (i) 200 µL BHI, (ii) 200 µL of sterilized deionized water, and (iii) a mixture of 100 µL of bacterial cells and 100 µL of sterilized deionized water. After 24 h incubation at 37 °C, 40 µL of INT was added and further incubated for 2 h before visualization for turbidity. All tests were done in triplicate. Ampicillin was employed as a positive control and the selected bacteria were wild types.

### 2.7. Antioxidant Activity

#### 2.7.1. Ferric Reducing Antioxidant Power (FRAP) Assay

The FRAP assay was conducted by adding 10 µL of the sample as well as the standard (ascorbic acid) to a 96-well plate [48]. Thereafter, 300 µL of the FRAP reagent (3 mL of iron (III) chloride hexahydrate + 3 mL of 2,4,6-tris(2-pyridyl)-s-triazine + 30 mL of acetate buffer + 6 mL of water) was added to all the wells. The reaction mixture was allowed to stand for 30 min at room temperature before the absorbance reading was taken at 593 nm. This assay was done in triplicate and the results were expressed as mM ascorbic acid equivalents per Gram (mM AAE/g).

#### 2.7.2. Folin–Ciocalteu (FC) Assay

The procedure of Salari et al. [49] was adopted with slight changes. Briefly, 25 µL of both the standard and the samples were added to a 96-well plate. The Folin–Ciocalteu’s phenol reagent (125 µL) was added followed by 100 µL of Na_2_CO_3_ solution. The plate was allowed to stand on the bench for 2 h at room temperature before the absorbance reading was taken at 765 nm. The experiment was conducted in triplicate and the results expressed mM gallic acid equivalents per Gram (mM GAE/g).

#### 2.7.3. 2,2′-Azino-bis-3-Ethylbenzotiazolin-6-Sulfonic Acid (ABTS) Assay

The method of Pu and co-workers [48] was adopted and slightly modified. In this experiment, 25 µL of the standard (Trolox) and samples were added to a 96-well plate followed by the ABTS reagent. The reaction mixture was allowed to stand for 30 min before the absorbance was read at 734 nm. The ABTS reagent was prepared 30 min before the experiment at 70 °C. The assay was repeated two more times. The results were expressed as mM Trolox equivalents per Gram sample (mM TE/g).

### 2.8. Statistical Analysis

The results of the bioactivities were analysed using two-way ANOVA followed by post hoc Tukey’s multiple comparisons test using GraphPad Prism software version 6.05 for Windows (GraphPad Software, La Jolla, CA, USA (www.graphpad.com)). The image analysis software ImageJ 1.50b version 1.8.0_60 (http://imagej.nih.gov/ij) and Origin pro 2019 64 bits were used to analyse the TEM and XRD images.

## 3. Results and Discussion

### 3.1. Identification and Mechanism of Procyanidin-AgNPs Formation

**Scheme 1:**
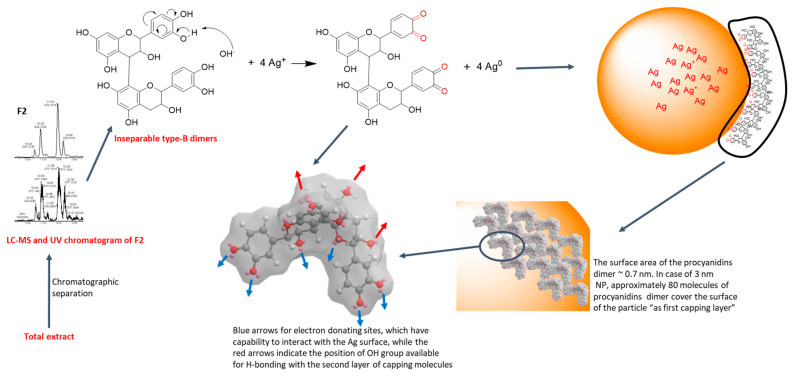
Proposed mechanism of procyanidin-mediated silver nanoparticles formation.

In our previous work, nuclear magnetic resonance (NMR) spectroscopy and liquid chromatography-mass spectrometry (LC-MS) aided the identification of F2, F1 and LSTE [40] contents. F1 showed a mixture of procyanidin dimers and trimers, while F2 showed a very pure isomeric structure of procyanidin dimers (type-B). On the other hand, the total extract (LSTE) showed in addition to procyanidins dimers and trimers, small flavonoids, and phenolic acids. Taking the B-type procyanidin dimers as an example (fraction F2), the mechanism of formation of AgNPs is demonstrated (Scheme 1). Following the green synthesis protocols, the starting materials were dissolved in ultrapure water. As water ionizes into protons and hydroxyl ions, the OH^-^ abstracts a proton from one of the hydroxyl groups on the compound (e.g., procyanidin B3) and an alkoxide ion is generated. The alkoxide species then reduces the silver ion (Ag^+^) to Ag^0^, thereby generating the NPs, and since the oxidation products depend on the alkoxide precursor, quinones are formed (Scheme 1) [50].

The proposed mechanisms were based on the following: Firstly, several polyphenols have been employed in Au- and AgNPs formation, the success of which has been ascribed to the presence of one or more hydroxyl groups [11,20,51,52]. Interestingly, procyanidins are poly-hydroxy compounds, confirming their suitability. In addition, to date, there has not been any report, as far as we know, on the successful fabrication of Au- or AgNPs using pure compounds without at least one hydroxyl functional group. Secondly, we hypothesized that alkoxide ions generated from the compounds are the chief reducing agents in the biosynthesis of AgNPs. This conclusion was made based upon potassium *tert*-butoxide being used as a reducing agent for the formation of Au and AgNPs through the green method. Upon the addition of *tert*-butoxide solution to gold and silver solutions, red and yellow colours appeared respectively [50]. Since the solution contained only K^+^ and *tert*-butoxide ion, it is evident that the latter was responsible for the reduction. Similarly, phenolate (a phenoxide) has also been used in the synthesis of AgNPs [53]. One of the advantages of this proposal is that all –OH carrying compounds can generate alkoxide ions before transforming to ketones. This can be more generalized, as all ‘reducing agents’ must possess at least one –OH group.

Further, most of the molecules employed as capping agents possess high molecular weights in addition to at least one electro-negative element or group such as –NH_2_, –COOH, –SH, –OH, and –CO. It is, therefore, hypothesized that groups such as –CO and –OH, being part of the precursors, must have provided such stability. This is because the NPs were stable without the use of any external stabilizers. The procyanidins dimers (and trimers) have great potential as antioxidant agents because of the phenolic hydroxyls and ease of transferring an electron and H^+^ to enzymes and/or metals. Because of the presence of stereogenic centres at C-2, C-3, and C-4, there are possible combinations for B-type dimers (different possibilities of interflavane bonds between C_α/β_-4 to C6/C_α/β_-4 to C-8). In addition, there are different conformers for each of the B-type dimers, however the extended (where B-rings are facing each other) and compact (where B-rings are opposite each other) are the most dominant. In the case of *O*-dioxy derivatives, the compact conformer has the minimum energy since the intra-hydrogen bonding doesn’t play an important role in the stabilization of the extended conformer [54]. Not all the procyanidin constituents were used in the formation of NPs. Therefore, the molecules in direct contact with the metal NPs, most probably are the dioxy derivatives. The second and even third capping layers can then be formed due to the inter-hydrogen bonding.

### 3.2. UV-Visibleible Analysis

**Figure 1:**
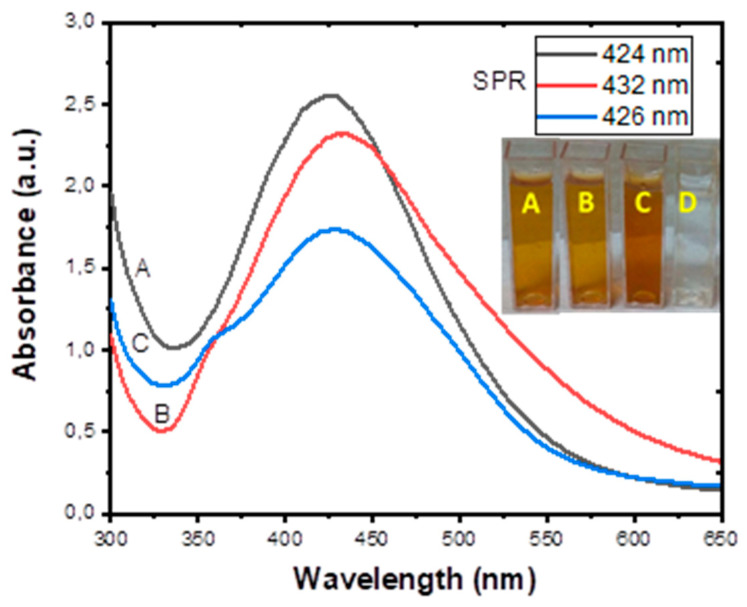
Absorption spectra of (**A**) *Leucosidea sericea* total extract-, (**B**) F1-, and (**C**) F2-mediated silver nanoparticles. The cuvettes labelled A-C (inset) represent the colour of the respective AgNPs while the clear solution (**D**) shows the colour of 1 mM AgNO_3_ solution used for the biosynthesis.

The result of UV-Visible analysis of (A) LSTE-, (B) F1-, and (C) F2-AgNPs are shown in Figure 1. The AgNPs exhibited surface plasmon resonance (SPR) bands between 424–432 nm, which confirmed the successful formation of the nanoparticles. A colour change was also observed within 10 min from yellowish to brownish (inset in Figure 1) when 1 mM silver nitrate (AgNO_3_) solution (D) was added, which indicated the successful synthesis of AgNPs. Hence, phytoconstituents in LSTE, F1, and F2, acted as reducing agents and thereafter as capping agents during the synthesis of the AgNPs. The similarity in the composition might have caused close SPR of the nanoparticles. No external capping agents were used, implying that the respective constituents served as both the reducing and capping agent. It is believed that polyphenols, abundantly available in plants, are responsible for the reduction of metal salts in NP synthesis [55]. In this case, procyanidins are undoubtedly responsible for the reduction and stabilization in both F1- and F2-AgNPs. The plasmonic resonance of AgNPs spans the range of 320–500 nm according to previous studies [56,57]. In this study, the SPR of the three AgNPs was observed to fall in the same range, which implies that the procyanidins with similar functional groups are the major contributing reducing and capping agents, even in the extract.

### 3.3. HRTEM Analysis

**Figure 2:**
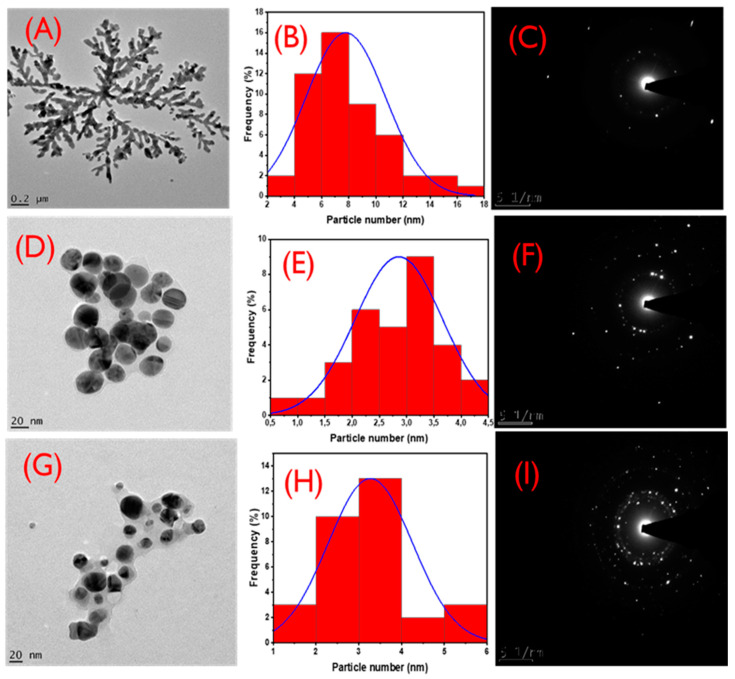
High-Resolution transmission electron microscopy images for *Leucosidea sericea* total extract-, F1-, and F2-mediated silver nanoparticles are represented as (**A**,**D**,**G**) and the corresponding particle size distributions as (**B**,**E**,**H**) respectively. The corresponding selected area electron diffraction pattern of the respective HRTEM images are represented as (**C**,**F**,**I**).

Figure 2A–F shows the HRTEM images, the corresponding particle size distributions, and SAED patterns of LSTE-, F1-, and F2-AgNPs. The average particle sizes were found to be 7.8 ± 2.9, 2.9 ± 0.8, and 3.3 ± 1.2 nm respectively. F1- and F2-AgNPs were more monodispersed and spherical in shape. Dendritic shapes, however, were observed for the LSTE-AgNPs (Figure 2A). This dendritic architecture has been previously reported [53,54]. Different shapes of silver NPs have been ascribed to a number of factors including the distance of formation to thermodynamic equilibrium and the fact that shape control for biological synthesis is still in its infancy [58]. Furthermore, spherical shapes were predominant for F1- and F2-AgNPs. The green synthesis of MNPs often results in nanoparticles of different shapes. The presence of a mixture of shapes such as irregular, triangular, hexagonal, and spherical is because of phytochemicals of different functional groups serving as reducing as well as stabilizing agents [59]. On the other hand, previous studies have shown that polyphenols in their pure forms often account for nanoparticles with spherical shapes, which were also observed in this study with F1- and F2-AgNPs [51,60]. Similar shapes and sizes have also been reported, in agreement with our findings [49,61]. Figure 2C,F,I depict the selected area electron diffraction (SAED) patterns of LSTE-, F1-, and F2-AgNPs respectively. The brightly circular patterns indicate that the particles are polycrystalline and can be indexed to the (111), (200), (220), and (311) planes of a face-centred cubic (FCC) structure of silver [51].

### 3.4. XRD Analysis

**Figure 3:**
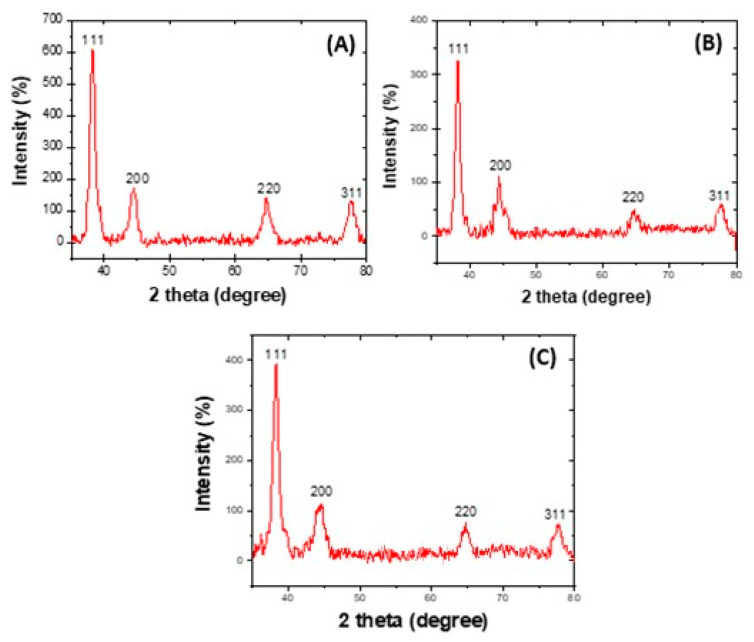
X-ray Diffraction patterns of the silver nanoparticles formed from *Leucosidea sericea* total extract (**A**), F1 (**B**), and F2 (**C**) showing the crystalline nature of the particles.

Figure 3 displays the XRD markings of the AgNPs. The 2θ (degree) values of 38.2, 44.4, 64.6, and 77.5 correlate orderly with the (111), (200), (220), and (311) planes of a face-centred cubic (FCC) silver lattice [62], in agreement with the standard silver structure (JCPS no. 04-0783). The XRD decoration also indicated that the biosynthesized silver nanoparticles are crystalline. Further supporting evidence of crystallinity are given by the presence of bright spots observed in the SAED patterns in Figure 2.

### 3.5. DLS Measurement

LSTE-, F1-, and F2-AgNPs show hydrodynamic sizes of 87.64, 95.17, and 148.80 nm respectively (Table 1). In most cases, smaller sizes are always recorded by TEM compared to the Zetasizer [63]. Although the size difference is often not more than two-fold, the literature records cases of wider difference. Siddiqi and colleagues [64] reported an average hydrodynamic size of 437.1 nm for AgNPs whereas the TEM measurement showed 9.40–11.23 nm. The vast difference in sizes obtained could be due to the different sampling volume used during the TEM and DLS measurements.

The polydisperity index (PDI), otherwise called the heterogeneity index, is the degree of non-uniformity of the size distribution of particles [65]. PDI is dimensionless and different size algorithms work with values between 0.05–0.7. According to the standard, PDI values of 0.05 and below are highly monodispersed. On the other hand, values greater than 0.7 shows broad particle size distribution [65]. In this context, the PDI is approximately 0.5, tending towards the extreme of 0.7 and further supporting the shape of F2-AgNPs. Therefore, LSTE- and F1-AgNPs possess more uniform particles and are stable to a similar extent. F2-AgNPs with larger sizes and a mixture of shapes have the tendency to aggregate faster owing to fewer capping agents. The zeta potential (ZP) is associated with charges around nanoparticles. When ZP is less than −30 mV, it means particles are mostly surrounded by negative charges. The magnitude of ZP determines the stability of the particles. Thus, the higher the value, the better the stability. The ZP of AgNPs indicates good stability [66]. The bimodal distribution of F2-AgNPs (Figure S1) is an indication that particles of different morphologies are present.

### 3.6. In Vitro Stability Study

As with the size and morphology, the stability of nanoparticles is of great significance for their application, especially in biological systems. Particles should retain their known properties even as they interact with cells and other biomolecules. As displayed in Figure S2, the UV-Visible measurement was termed 0 h (A1, B1, C1) upon the addition of various solutions to the respective AgNPs. Aqueous, saline, protein, and buffer solutions interacted with F1-, F2-, and LSTE-AgNPs. The absorbance indicates that all solutions interacted similarly with the NPs, except BSA. The chemical structure and reaction of BSA differ from those of glycine and cysteine. It has been reported that BSA possesses about 50 lysines on its surface, rendering it to show a high affinity for negative surfaces [67]. Interestingly, the AgNPs in this study are surrounded by negative ions as suggested by negative ZP values. Therefore, this results in an enhanced interaction between the AgNPs and BSA. Although the interaction lowers after 48 h in the case of F1 and LSTE, it was still high for F1 at the same time point. This may also be due to a slightly higher ZP value of F1 compared to others. By 24 h, changes became more visible by other solutions. The blue or redshifts were minimal for F1 (A2) and LSTE (C2) but more pronounced for F2 (B2). This behaviour could be attributed to the homo- or heterogeneity of the NPs. PDI values of 0.393 and 0.398 were recorded for F1- and LSTE-AgNPs respectively, while 0.472 was observed for F2. This implies that F1- and LSTE-AgNPs were relatively more uniform in shape than the F2-AgNPs [65]. This trend continued in the 48-hr measurement. At this time, even the F1 and LSTE-AgNPs displayed slight variations. It could be observed that the extent of segregation of the absorbance peaks had also increased in the F1-AgNPs such that the stability of the NPs is in the order of LSTE > F1 > F2. This may be due to the abundance of phytochemicals involved in the formation and encapsulation of LSTE-AgNPs. The temperature of 37 °C, the biological media, and physiological pH were selected to mimic the human body conditions. If the particles interacted with the chosen molecules and retain their characteristic features at such conditions, it can be inferred that further biological studies are necessary. Thus, the evaluation confirms that they can interact with cells without losing their properties.

### 3.7. Dilution Study

**Figure 4:**
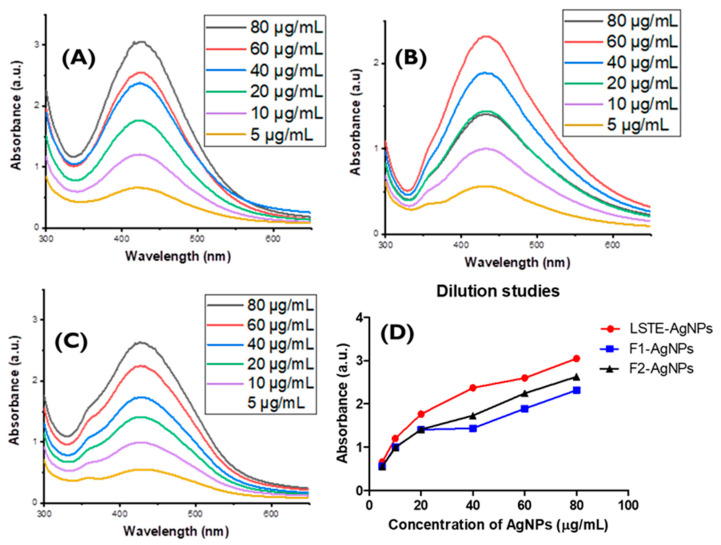
Surface Plasmon Resonance (λ max) of (**A**) *Leucosidea sericea* total extract mediated silver nanoparticles, (**B**) F1-mediated silver nanoparticles, and (**C**) F2-mediated silver nanoparticles showing the retention of properties by the particles even at low concentrations. Dilution studies (**D**) showing the linearity of diluted concentration.

Since certain biological applications may require different concentrations of AgNPs, dilution studies are necessary to confirm the retention of properties before future utilization [40]. Figure 4 shows the UV-Visible results of (A) LSTE-, (B) F1-, and (C) F2-AgNPs. The absorption spectra obtained for the different concentrations of the nanoparticles (80, 60, 40, 20, 10, and 5 µg/mL) suggest that the SPR wavelengths are almost identical for all the solutions. This means that the dilution of nanoparticles does not affect the properties of LSTE-, F1-, and F2-AgNPs [40]. Furthermore, the study of intensity versus concentration affirmed a proportional relationship, as depicted in Figure 4D. The importance of this dilution study is in the dosage to be used for certain applications. For the prepared nanoparticles to be active at any given concentration, their physicochemical features need to be retained. One of the quickest ways to confirm this is through their absorbance in UV-Visible spectroscopy, a powerful tool in green synthesis. Thus, if there was no absorbance at the expected region, it may be inferred that the particles have lost their properties and may not be useful at such concentration. This serves as a guide for subsequent applications and consideration of another principle of green chemistry (atom economy).

### 3.8. In-Vitro Enzyme Inhibition

**Figure 5:**
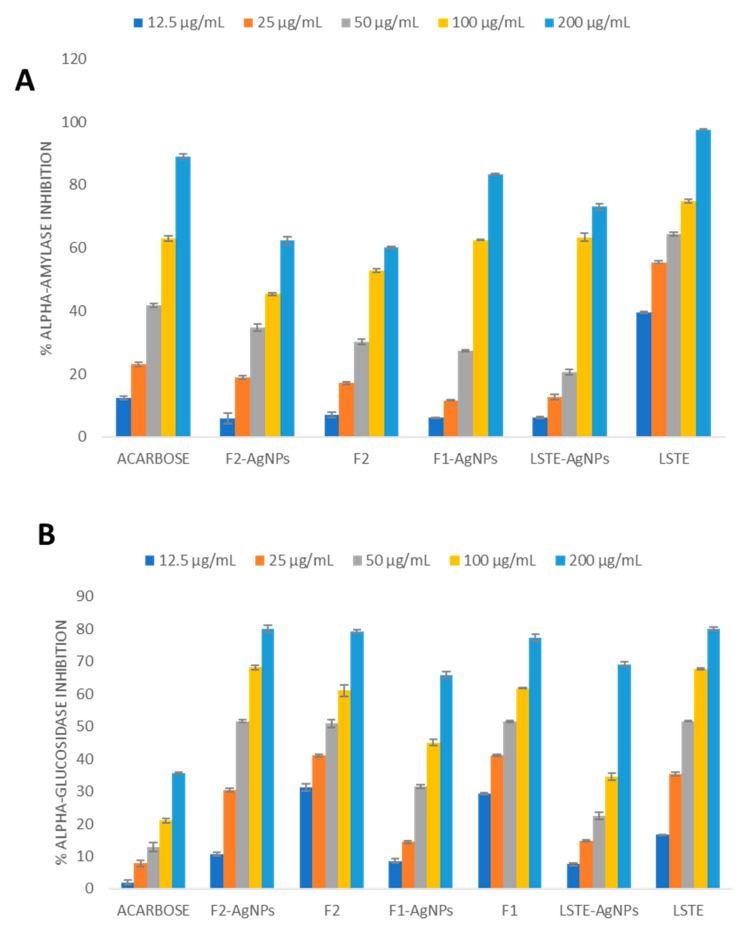
Antidiabetic activities with regards to alpha-amylase (**A**) and alpha-glucosidase (**B**) inhibition by *Leucosidea sericea* total extract (LSTE), procyanidin fractions (F1 and F2), and their respective silver nanoparticles (LSTE-, F1-, and F2-AgNPs).

Table 2 depicts the enzyme inhibitory IC_50_ values of the three AgNPs alongside their corresponding intact fractions. Alpha-glucosidase inhibitory IC_50_ values of 21.48 and 18.76 µg/mL were recorded for LSTE- and F1-AgNPs, respectively. These activities imply that the AgNPs possess potent inhibitory characteristics when compared to the standard drug, acarbose. However, when considering F2-AgNPs, it showed a similar inhibition activity as that of its precursor (F2), perhaps because of the remains of procyanidins on the surface of the nanoparticles as capping agents. On the other hand, LSTE- and F1-AgNPs displayed moderate alpha-amylase inhibitory activity in comparison with acarbose even though LSTE still showed a very high inhibition when compared to its corresponding NPs. Interestingly, F1-AgNPs displayed improved alpha-amylase inhibitory activity when compared to its intact fraction, F1, displaying no inhibition. The percentage inhibition at various concentrations was presented in Figure 5.

As stated earlier for alpha-glucosidase, F2-AgNPs also showed a similar IC_50_ value as its precursor (F2) for alpha-amylase. This may be linked to the unique characteristics of these NPs including its morphology. It further shows that the activity may be dependent on size, shape, and other factors such as the type of phytochemical involved as the reductant and capping agent [46]. Previously, AgNPs have displayed alpha-glucosidase inhibition [30]. Our results are also supported by previous antidiabetic studies of AgNPs [30,31,68]. In summary, the AgNPs in this study demonstrated interesting enzymatic activity, although mostly lower than those of the corresponding precursors, the most important fact is that they showed inhibitory activities.

### 3.9. Antioxidant Activity

**Figure 6:**
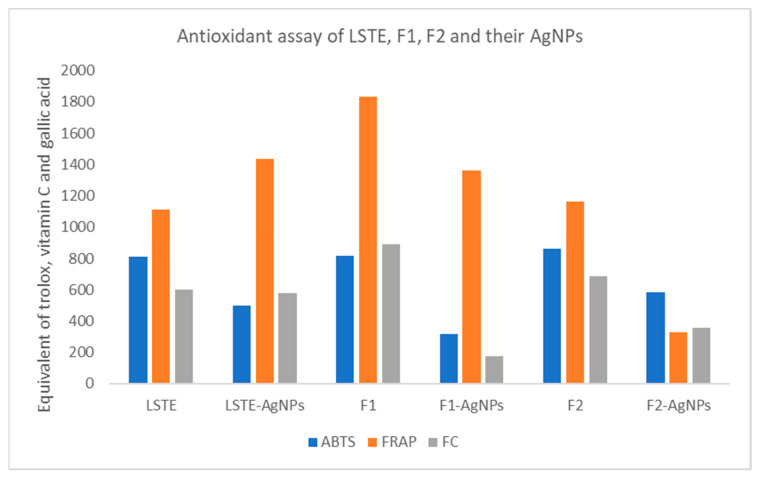
Antioxidant activity in terms of the ABTS (2,2′-azino-bis-3-ethylbenzotiazolin-6-sulfonic acid), FRAP (ferric reducing antioxidant power), and FC (Folin–Ciocalteu) scavenging activity by *Leucosidea sericea* total extract (LSTE), procyanidin fractions (F1 and F2), and their respective silver nanoparticles (LSTE-, F1-, and F2-AgNPs). The antioxidant activities were measured based on the equivalence of standard antioxidants Trolox, vitamin C (ascorbic acid), and gallic acid.

Antioxidants possess free radical scavenging abilities. Table 3 reports the scavenging ability of the three AgNPs biosynthesized from LSTE, F1, and F2 and the respective intact fractions. The antioxidant activities, as measured by the FRAP, ABTS, and FC assays, of the intact fractions were significantly (*p* < 0.01) higher than those of their corresponding AgNPs. For instance, when considering the ABTS assay, all the fractions, roughly showed a doubling of antioxidant activity when compared to the hybrid nanoparticles. This could be ascribed to a change in the functional groups during the nanoparticle formation, rendering them unavailable to participate in the scavenging activity with a subsequent reduction in the antioxidant activity. The antioxidant activity as the equivalence of the standard (Trolox, vitamin C, and gallic acid) for ABTS, FRAP, and FC assays respectively, has been shown in Figure 6.

When considering the FRAP assay outcomes, a similar trend was observed for the NPs and their corresponding intact fractions, F1 and F2, but not for LSTE, which showed increased antioxidant activity for its corresponding NP. On the other hand, when considering the FC assay outcomes, similar antioxidant activities were observed for LSTE and its corresponding NP, but for F1 and F2, their corresponding NPs showed a significantly lower antioxidant activity.

The significantly higher antioxidant activity displayed by F2 vs. the F2-AgNPs could be due to low stability, size, and heterogeneity of the latter. Evidently, LSTE-AgNPs displayed better activity than F1- and F2-AgNPs and competes well with the intact fractions. This may be due to the abundance of the phytochemicals surrounding the LSTE nanoparticles as capping agents [49]. Previously, it has been suggested that the activity of nanomaterials may also be a function of the capping agents [46]. Our results were also in agreement with the activity reported by [49]. Reports have linked antioxidants to many deadly diseases including diabetes, hence, materials with dual properties would be of great benefit in reducing these menaces. A herbal formulation [69], the extracts of *Ananas comosus* [30], and *Chamaecostus cuspidatus* [55] were previously employed in synthesizing NPs with encouraging antidiabetic and antioxidant activities. Since certain antioxidants may also be implicated in adverse health-effects, several studies have been done on the antioxidant properties of many biosynthesized nanomaterials in the quest to finding more suitable alternatives [48,70,71].

### 3.10. The Antibacterial Assay of AgNPs

Silver nanoparticles have been in the lead in terms of antibacterial activities [72]. When various plant parts were used for the synthesis, AgNPs have shown potent bactericidal effects on *E. coli* and *S. aureus* [72]. In the current study, the antibacterial activities of the AgNPs were also studied using wild species of both Gram-positive and Gram-negative bacteria. Serially diluted concentrations of the AgNPs were used and the results showed that the MIC values were in the range of 15.63–125 µg/mL [67].

Bacterial growth inhibition varied between the AgNPs tested. Among the three AgNPs, the MIC value of 62.50 μg/mL was most common (Table 4). This was particularly the case with F2-AgNPs except for *B. cereus* where the MIC was at the highest concentration of 125 μg/mL. This was still an improvement as none of the fractions showed any activity even above 1000 μg/mL. Similarly, nanoparticles (NPs) with similar sizes and MICs have been reported [73]. Research has shown that the activity of AgNPs is greatly associated with the concentration, shape, and size of the NPs [72,74], and that AgNPs of small sizes at low concentrations are often effective antibacterial agents [75]. Various researchers evaluated the activity of these NPs with different sizes and shapes against certain species of bacteria [74,76,77] and found that 2.5–85 nm-sized AgNPs showed antibacterial activities, while the most effective ones appeared to be between 2.5–20 nm. This implies that smaller silver nanoparticles are highly effective antibacterial agents. In addition, other authors reported that the activities of AgNPs may not only be due to small size but the overall morphology. While Shao et al. showed that the enhanced antimicrobial activity was due to the small size and spherical shape of their particles, Sahu and colleagues implicated small size and mono-dispersity over large and polydispersed ones [78,79]. From our TEM results, the average size was determined to be in the range of 2.9–7.8 nm. Consequently, LSTE-AgNPs presented the lowest bactericidal activity. Although its MIC falls mostly at 62.50 µg/mL, the percentage inhibitions were only moderate for *P. aeruginosa* and *S. aureus* respectively. The *B. cereus* displayed a mild inhibition whereas *S. marcescens* were completely wiped out at the same concentration. This was, however, not far-fetched as the heavy presence of phenolic content was still observed, evidenced by the values presented in Table 3. It should be noted, however, that the intact fractions did not show any activity even at high concentrations (2000 µg/mL). On the other hand, the bactericidal effect of the AgNPs depends on the type of bacteria. In general, the Gram-negative bacterial species responded better to the bactericidal effects of the AgNPs than the Gram-positive species. Previous researchers have also shown this. It has been explained that the Gram-positive bacterial species possess thicker cell walls which makes penetration more difficult. The thinner cell wall of the Gram-negative species may have allowed easier interaction and possible disruption leading to more activity [80]. Overall, the bactericidal effects of our AgNPs appeared to be size-dependent. The smallest AgNPs (2.9 nm) presented more bactericidal effects in four organisms. This includes; *P. aeruginosa*, *B. cereus*, *E. coli*, with MIC of 31.25 μg/mL and even as low as 15.63 μg/mL for *S. aureus*. In a similar study, the antibacterial activity of AgNPs on *S. aureus* at 16.12 μg/mL has been reported [81]. When the particles are smaller, a larger surface area is available for contact with the bacteria, which could possibly lead to greater interaction with the bacteria. Therefore, F1-AgNPs had the largest contact with the bacteria, and might possibly explain the enhanced antibacterial activities observed [82]. Furthermore, previous studies have shown that the antimicrobial activity of a series of AgNPs increased as the size decreased [83]. Our findings, therefore, agree with these studies.

Finally, extensive research activities are on-going in the field of green nanotechnology. Several authors have reported biosynthesis as a better procedure of forming nanoparticles while maintaining the principles of green chemistry. This study and many others, as mentioned previously, showed the potential of AgNPs as antibacterial agents and can form an important combination with antibiotics against drug-resistant micro-organisms. On the other hand, the previous toxicity measurements indicated marginal toxic effects of AgNPs in vitro [32,33]. In vivo studies are very limited but indicated toxicity in rats. The toxicity of AgNPs largely depends on the surface charges and the stabilizing (capping) agents which control the cells uptake of the NPs. A changing surface structure of AgNPs may lead to safe AgNPs for human uses and also increase the bioactivity.

Further studies are required to investigate the effects of procyanidins as capping agents and other natural compounds on the activity, cell uptake, and toxicity of the AgNPs. The future in vivo studies are highly appreciated in this regard and considered to be essential and helpful to understand the efficiency and safety of the prepared AgNPs utilizing edible, safe, and active compounds such as procyanidins. In addition, investigation of the mechanism of action of AgNPs as a key factor is yet to be established using different in vitro studies.

## 4. Conclusions

To our knowledge, this represents the first scientific research pertaining to the use of *Leucosidea sericea* constituents for AgNPs fabrication and subsequent evaluation of their in vitro anti-diabetic, antioxidant, and anti-bacterial activities. Capping agents play important roles in determining the final characteristics of the metal NPs. The procyanidin dimers (type-B) were isolated and employed to synthesise stable and bioactive AgNPs. The results are very encouraging and showed great enhancement in the activities of the intact fractions. The natural products/nanoparticles combination is a new important dimension in the area of drug discovery and the use of natural product compounds, such as procyanidins, that have well-established pharmacological profiles will improve the future usage of the metal NPs in the field of biomedical applications.

## Figures and Tables

**Table 1:** Particle size and zeta potential for AgNPs obtained from Dynamic Light Scattering.

Items	Hydrodynamic Size (nm)	Polydisperity Index	Zeta Potential (mV)
LSTE-AgNPs	87.64	0.398	−25.7
F1-AgNPs	95.17	0.393	−29.4
F2-AgNPs	148.80	0.472	−28.8

**Table 2:** The enzymatic inhibitory activity, expressed as IC_50_ of the *Leucosidea sericea* total extract (LSTE), the intact fractions (F1 and F2), and those of their corresponding silver nanoparticles (LSTE-, F1- and F2-AgNPs) using two assays (µg/mL).

Items	Alpha-Amylase (IC_50_) (µg/mL)	Alpha-Glucosidase (IC_50_) (µg/mL)
LSTE	3.50 ± 0.70 ^a^	8.10 ± 0.60 ^a^
LSTE-AgNPs	14.92 ± 1.0 ^b^	21.48 ± 0.90 ^b^
F1	NA	7.30 ± 0.50 ^a^
F1-AgNPs	13.24 ± 0.60 ^b^	18.76 ± 1.00 ^c^
F2	18.9 ± 0.20 ^c^	7.10 ± 0.40 ^a^
F2-AgNPs	19.13 ± 0.80 ^c^	8.75 ± 0.70 ^a^
Acarbose	10.20 ± 0.40 ^d^	61.00 ± 1.50 ^d^

The results are expressed as mean ± SD of three different independent experiments (*n* = 3). Results not sharing a common superscript alphabet (^a–d^) are significantly different (*p* < 0.01). Acarbose (standard), NA (not active at the tested concentrations).

**Table 3:** The antioxidant activity of the *L. sericea* total extract (LSTE), the intact fractions (F1 and F2), and those of their corresponding silver nanoparticles (LSTE-, F1-, and F2-AgNPs) using three assays (ABTS, FRAP, and FC). The percentage phenolic content (FC) of the silver nanoparticles is also presented in the last column of the table.

Items	ABTS (mM TE/g)	FRAP (mM AAE/g)	FC (mM GAE/g)	FC % (AgNPs)
LSTE	814.18 ± 1.80 ^a^	1113.20 ± 6.70 ^a^	602.60 ± 6.10 ^a^	100
LSTE-AgNPs	499.65 ± 1.50 ^b^	1438.50 ± 5.60 ^b^	578.27 ± 7.70 ^b^	57.8
F1	818.20 ± 7.70 ^a^	1834.00 ± 4.70 ^c^	889.60 ± 6.00 ^c^	100
F1-AgNPs	319.18 ± 1.80 ^c^	1361.60 ± 6.70 ^d^	175.25 ± 2.60 ^d^	17.5
F2	861.90 ± 5.30 ^d^	1166.00 ± 2.10 ^e^	685.70 ± 6.70 ^e^	100
F2-AgNPs	583.22 ± 7.30 ^e^	326.20 ± 2.20 ^f^	357.80 ± 5.30 ^f^	35.7

The results are expressed as mean ± SD of three different independent experiments (*n* = 3). Results not sharing a common superscript alphabet (^a–f^) are significantly different (*p* < 0.01).

**Table 4:** The minimum inhibitory concentration (MIC, µg/mL) values of the *L. sericea* total extract (LSTE), the intact fractions (F1 and F2), and their corresponding silver nanoparticles (LSTE-, F1- and F2-AgNPs) using six bacterial species.

Bacteria	LSTE	LSTE-AgNPs	F1	F1-AgNPs	F2	F2-AgNPs	Control *
*P. aeruginosa*	>2000	62.50	>2000	31.25	>2000	62.50	31.25
*S. aureus*	>2000	62.50	>2000	15.63	>2000	62.50	15.63
*B. cereus*	>2000	62.50	>2000	31.25	>2000	125.00	7.81
*S. enterica*	>2000	31.25	>2000	31.25	>2000	62.50	7.81
*E. coli*	>2000	62.50	>2000	31.25	>2000	62.50	15.63
*S. marcescens*	>2000	125.00	>2000	62.50	>2000	62.50	3.90

* Ampicillin.

## Supplementary Materials

The following are available online at https://www.mdpi.com/2079-4983/11/3/66/s1, Figure S1: Hydrodynamic size of (a) LSTE-, (c) F1-, (e) F2-AgNPs and zeta potential of (b) LSTE-, (d) F1- and (f) F2-AgNPs as measured by Dynamic Light Scattering technique, Figure S2: Stability of F1-AgNPs for (A1) 0 h, (A2) 24 h, (A3) 48 h, F2-AgNPs for (B1) 0 h, (B2) 24 h and (B3) 48 h and LSTE-AgNPs for (C1) 0 h, (C2) 24 h, (C3) 48 h in different solutions.
