# Supplementary material for: Biosynthesis, Characterization, and Biological Activities of Procyanidin Capped Silver Nanoparticles
Source: J Funct Biomater. 2020 Sep 19;11(3):66. doi: 10.3390/jfb11030066 (PMC7564108; doi:10.3390/jfb11030066)
Supplement: Supplementary file 1 [file jfb-11-00066-s001.pdf]

Supplementary File

Biosynthesis, Characterization, and Biological Activities of Procyanidin Capped Silver Nanoparticles

Umar M. Badeggi [1,†], Jelili A. Badmus [2,‡], Subelia S. Botha [3], Enas Ismail [1,#], Jeanine L. Marnewick [2], Charlene W. J. Africa [4] and Ahmed A. Hussein [1,*]

[1] Department of Chemistry, Cape Peninsula University of Technology, Symphony Rd. Bellville 7535, South Africa; 217064221@mycput.ac.za (U.M.B.); enas.ismail4@yahoo.com (E.I.)

[2] Applied Microbial and Health Biotechnology Institute, Cape Peninsula University of Technology, Symphony Rd. Bellville 7535, South Africa; jabadmus@lautech.edu.ng (J.A.B.); marnewickj@cput.ac.za (J.L.M.)

[3] Electron Microscope Unit, University of the Western Cape, Bellville 7535, South Africa; subotha@uwc.ac.za

[4] Department of Medical Biosciences, University of the Western Cape, Bellville, 7535, South Africa; cafrica@uwc.ac.za

[†] Permanent address: Department of Chemistry, Ibrahim Badamasi Babangida University Lapai, PMB 11, Minna 4947, Nigeria

[‡] Permanent address: Department of Biochemistry, Ladoke Akintola University of Technology, Ogbomoso, 210214 Nigeria

[#] Permanent address: Physics Department, Faculty of Science (Girls branch), Al Azhar University, Nasr city, Cairo 11884, Egypt

[*] Correspondence: mohammedam@cput.ac.za; Tel.: +27-21-959-6193; Fax: +27-21-959-3055

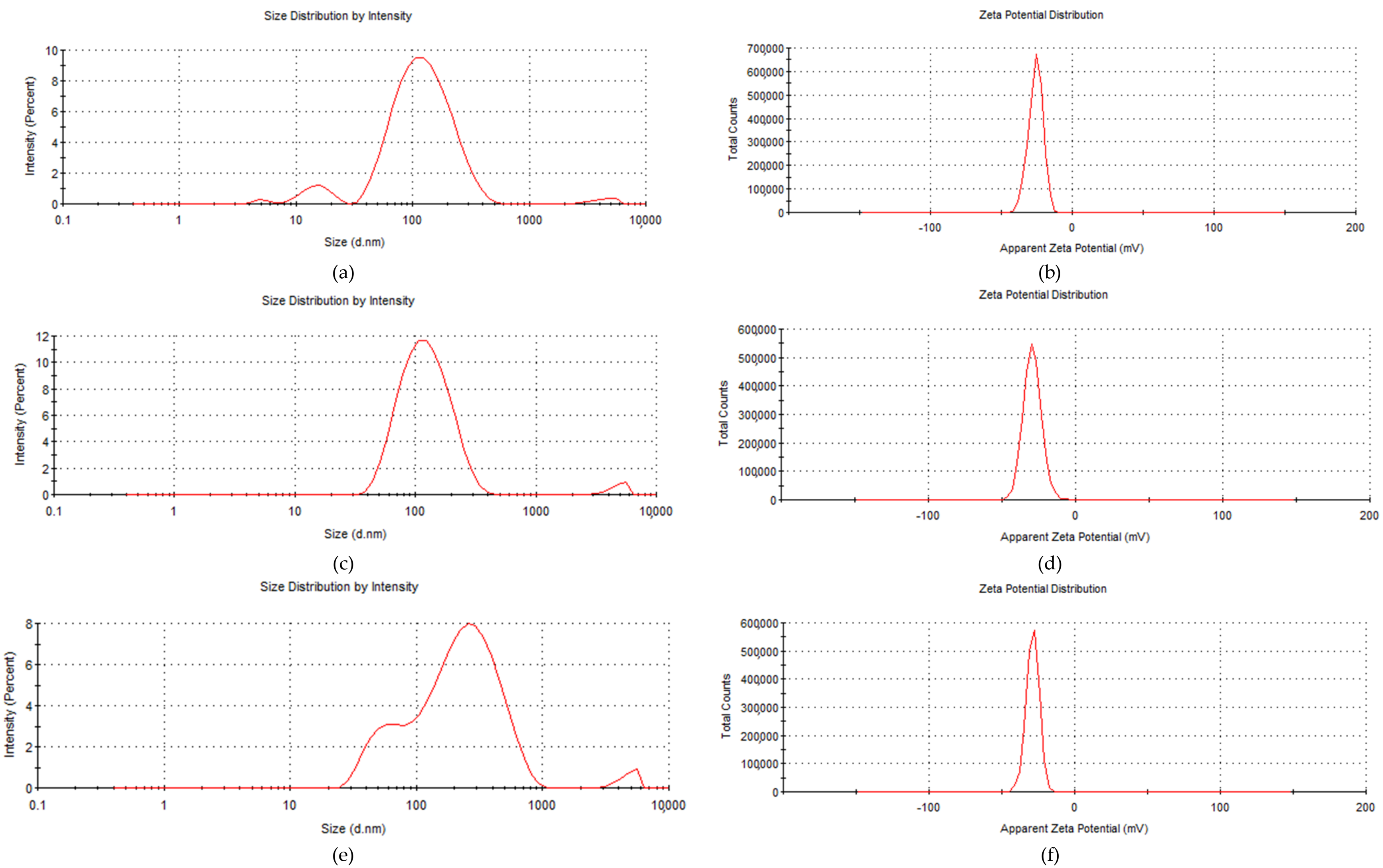

Figure S1. Hydrodynamic size of (a) *Leucosidea sericea* total extract-, (c) F1-, (e) F2-mediated silver nanoparticles and zeta potential of (b) *Leucosidea sericea* total extract, (d) F1- and (f) F2- mediated silver nanoparticles as measured by Dynamic Light Scattering technique.

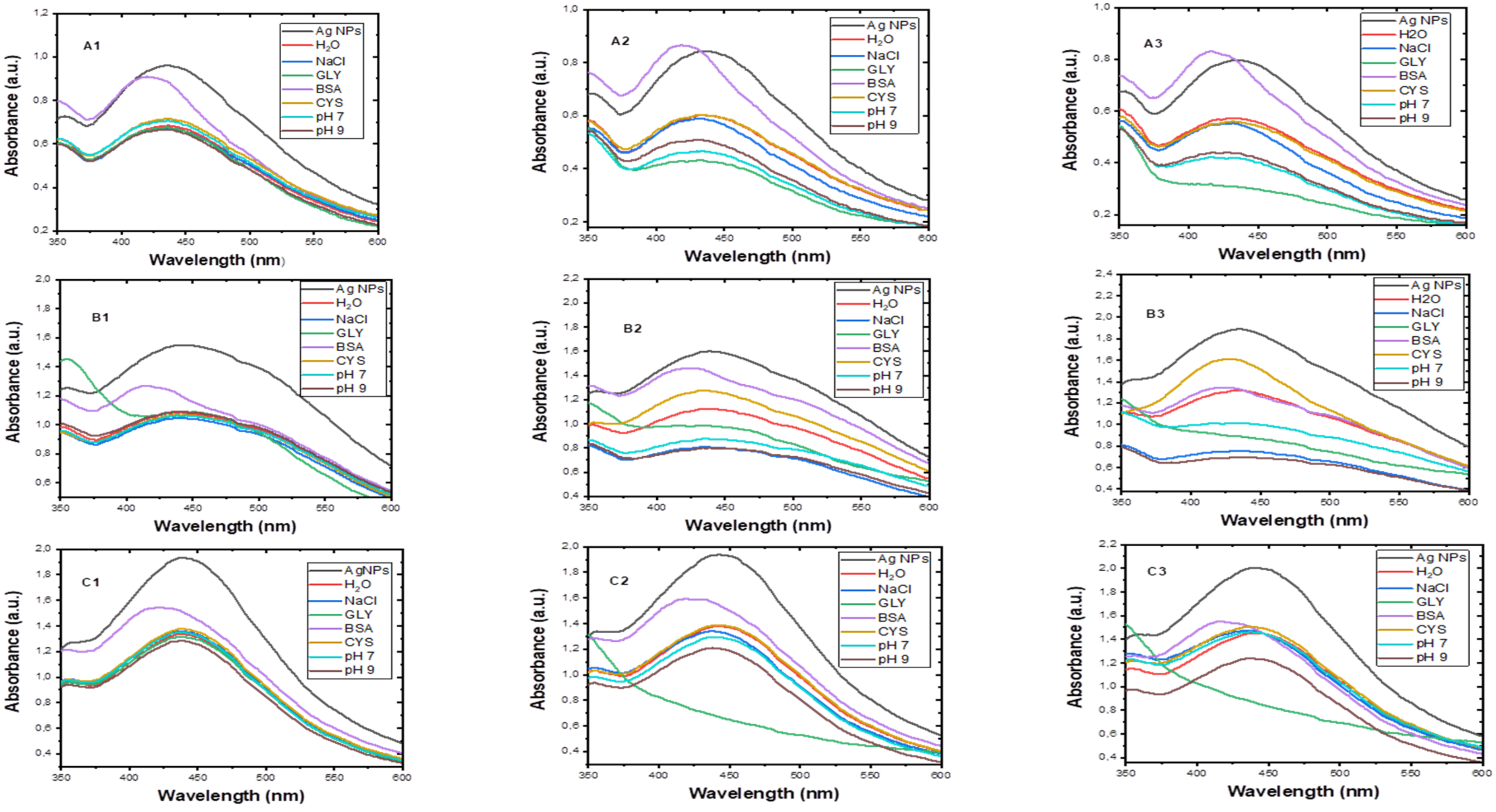

Figure S2. Stability of F1-mediated silver nanoparticles for (A1) 0 h, (A2) 24 h, (A3) 48 h, F2- mediated silver nanoparticles for (B1) 0 h, (B2) 24 h and (B3) 48 h and *Leucosidea sericea* total extract-mediated silver nanoparticles for (C1) 0 h, (C2) 24 h, (C3) 48 h in different solutions and buffers.
